# Impact of Fetuin-A (AHSG) on Tumor Progression and Type 2 Diabetes

**DOI:** 10.3390/ijms19082211

**Published:** 2018-07-29

**Authors:** Josiah Ochieng, Gladys Nangami, Amos Sakwe, Cierra Moye, Joel Alvarez, Diva Whalen, Portia Thomas, Philip Lammers

**Affiliations:** 1 Departments of Biochemistry, Cancer Biology, Neuroscience and Pharmacology, Meharry Medical College, 1005 D.B. Todd Blvd., Nashville, TN 37208, USA; gsimiyu@mmc.edu (G.N.); asakwe@mmc.edu (A.S.); cnvmoye@gmail.com (C.M.); jalvarez17@email.mmc.edu (J.A.); dwhalen15@email.mmc.edu (D.W.); pthomas13@email.mmc.edu (P.T.); 2 Departments of Internal Medicine, Meharry Medical College, 1005 D.B. Todd Blvd., Nashville, TN 37208, USA; plammers@mmc.edu

**Keywords:** fetuin-A, exosomes, tumor, attachment, growth

## Abstract

Fetuin-A is the protein product of the *AHSG* gene in humans. It is mainly synthesized by the liver in adult humans and is secreted into the blood where its concentration can vary from a low of ~0.2 mg/mL to a high of ~0.8 mg/mL. Presently, it is considered to be a multifunctional protein that plays important roles in diabetes, kidney disease, and cancer, as well as in inhibition of ectopic calcification. In this review we have focused on work that has been done regarding its potential role(s) in tumor progression and sequelae of diabetes. Recently a number of laboratories have demonstrated that a subset of tumor cells such as pancreatic, prostate and glioblastoma multiform synthesize ectopic fetuin-A, which drives their progression. Fetuin-A that is synthesized, modified, and secreted by tumor cells may be more relevant in understanding the pathophysiological role of this enigmatic protein in tumors, as opposed to the relatively high serum concentrations of the liver derived protein. Lastly, auto-antibodies to fetuin-A frequently appear in the sera of tumor patients that could be useful as biomarkers for early diagnosis. In diabetes, solid experimental evidence shows that fetuin-A binds the β-subunit of the insulin receptor to attenuate insulin signaling, thereby contributing to insulin resistance in type 2 diabetes mellitus (T2DM). Fetuin-A also may, together with free fatty acids, induce apoptotic signals in the beta islets cells of the pancreas, reducing the secretion of insulin and further exacerbating T2DM.

## 1. Introduction

Fetuin-A is a glycoprotein that is synthesized by a number of fetal tissues, while in adult animals including humans, it is synthesized mainly by the liver parenchyma cells [1]. Even though it is currently considered to be a multi-functional protein [2], its roles in disease processes such as diabetes [3,4,5] and kidney disease [6,7] as well as its ability to inhibit ectopic calcification [8] have gained the most mileage so far. The potential role of fetuin-A in tumor progression stemmed from earlier studies that suggested that it was the cell attachment factor in serum [9]. However, this role has been controversial ever since the glycoprotein was first isolated, purified, and characterized from bovine serum in 1944 [10].

Fetuin-A can be isolated and partially purified by adding ammonium sulfate to fetal bovine serum [11]. The resulting precipitate that is lyophilized after dialysis is commonly referred to as Pedersen fetuin-A. In most cases, Pedersen fetuin-A can replace Fetal Bovine Serum (FBS) as medium supplement to support the attachment and growth of a number of cells in vitro. In some cases, further purification strategies starting with Pedersen fetuin-A yielded more homogenous fetuin-A preparations that lacked cell attachment and growth promoting properties [12]. Specifically the Spiro method of purification employed zinc acetate and resulted in a single band ~48 kDa in SDS-PAGE gels that lacked biological activity. These studies provided evidence that attachment and growth-promoting properties of Pedersen fetuin-A were due to the contaminating protein factors that co-purified with fetuin-A. Subsequent studies demonstrated that incubation of fetuin-A (derived from fetal bovine serum) with Zn^2+^ ions as in the Spiro method, transformed the protein into an apoptosis-inducing factor, particularly in tumor cells [13]. Zinc ions at low concentrations such as in 5–10% (*v*/*v*) fetal bovine serum used to supplement growth media, promote cell growth in culture due to the fact that at these low concentrations, zinc act as an insulin mimetic [14,15]. However, at higher concentrations of Zn^2+^ (~500 µM) that are commonly used in fetuin-A purification buffers, zinc is toxic to cells, particularly when it is associated with fetuin-A in serum [16].

Pedersen fetuin-A co-purifies with a number of serum proteins such alpha 2 macroglobulin [17]. We have described a purification protocol where we subjected the Pedersen fetuin-A to a glycerol gradient centrifugation and obtained a final product that was >99% pure fetuin-A as determined by colloidal Coomassie blue staining of SDS-PAGE-gels [17]. Interestingly, fetuin-A purified according to this protocol retained the cell attachment and growth promoting properties of Pedersen fetuin-A, suggesting that the attachment and growth properties of fetuin-A should not be attributed to contaminating factors after all. The mechanisms mediated by the fetuin-A purified by the glycerol gradient centrifugation involved the activation of PI3 kinase-AKT, as well as MAP kinase signaling pathways [17]. This review will therefore focus on the involvement of fetuin-A in physiological processes that are germane to tumor growth and metastasis, both in vitro and in vivo.

Regarding its role in diabetes, fetuin-A is known to bind to the tandem fibronectin type 3 domains present in the extracellular portion of the transmembrane β-subunit of the insulin receptor (InsR), further away from the high-affinity pocket between the two α-subunits which comprise the binding site of insulin [18]. Interestingly only two proteins directly interact with the extracellular portion of the insulin receptor, i.e., insulin and fetuin-A. Insulin turns on the receptor’s intrinsic tyrosine kinase activity responsible for glucose transport. Fetuin-A on the other hand, turns off tyrosine kinase activity [18]. Thus even though fetuin-A does not bind to the same site as insulin on the extracellular portion of InsR, it attenuates insulin signaling, thereby contributing to insulin resistance. This relationship between insulin and fetuin-A is important for the control of glucose homeostasis. In addition to its regulation of insulin signaling at the receptor level, fetuin-A also modulated FFA-mediated inflammation of the β-cells in the pancreas [19], thereby contributing to insulin resistance at this level also. We will discuss these two pathways by which fetuin-A mediates insulin resistance, thereby contributing to the sequelae of type 2 diabetes and pre-diabetes.

## 2. Structural Features of Fetuin-A

(AHSG) is the human homologue of the protein that was first described in bovine serum as fetuin. It is a 55–59 kDa phosphorylated glycoprotein synthesized by the liver hepatocytes. For the sake of simplicity we will refer to it as fetuin-A throughout this review, to distinguish it from its paralogue fetuin-B [20,21].

**Figure 1:**
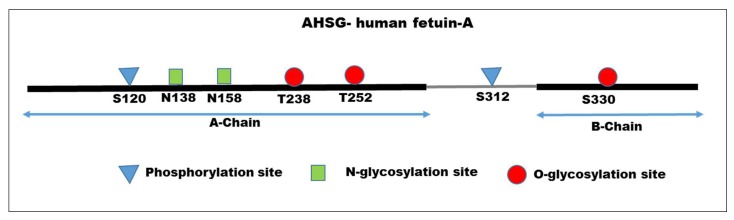
Amino acid sequence of human fetuin-A. The mature form of AHSG consists of A-chain (residues 1–282); connecting peptide (grey line) residues 283–321; B-chain residues 323–349. There are two phosphorylation sites (residues S120 and S312); two *N*-glycosylation sites (N138, N158) and two *O*-glycosylation (T238, T252) sites in the A-chain. There is one *O*-glycosylation site in the B-chain (S330).

The human and bovine forms of fetuin-A both share 12 conserved cysteine residues where the circulating protein has six disulfide bonds [22]. Interestingly, the dominant form of human fetuin-A can be processed by proteases to a large A-chain (residues 19–300) connected to a smaller B-chain (residues 341–367) via a single disulfide linkage, and a 40-residue-connecting polypeptide is lost during processing [23]. Later refinements clarified phosphorylation and glycosylation sites in the protein (Figure 1). There are two phosphorylation sites, one in the A-chain and the other in the connecting peptide. There are two *N*-glycosylation sites and two *O*-glycosylation sites in the A-chain, and one *O*-glycosylation site in B-chain [24,25]. Interestingly, orthologues of fetuin-A are found in 47 vertebrates ranging from fish to mammals. All of the species show conserved 12 Cys residues, clearly indicating that the disulfide bonds formed dictate the three dimensional structure of fetuin-A. Fetuin-A is a member of the cystatin family of proteins. There are two tandem cystatin-homology domains in all vertebrate fetuin-A [26]. Interestingly, close to 20% of serum fetuin-A is phosphorylated (Ser312 in the mature protein), but the physiological role of the phosphorylated protein is yet to be defined [25,27,28]. Fetuin-A is located on chromosome 3q27 [29].

## 3. Role of Fetuin-A in Cell Attachment, Motility, and Invasion of Tumor Cells

**Figure 2:**
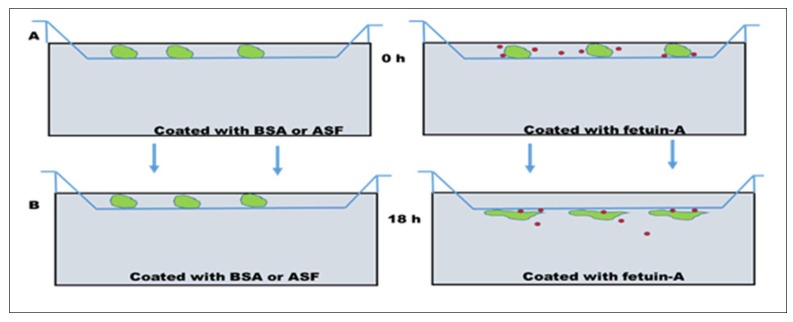
Model depicting how fetuin-A mediates motility and adhesion in tumor cells. (**A**), the lower wells of a Boyden Chamber plate can be coated with either bovine serum albumin (BSA) or asialofetuin-A (ASF) as a control, or with fetuin-A. Due to on and off rates, the coated fetuin-A can move by simple diffusion to the upper chambers containing the cells, enter the cells (green) on the top wells, and promote the secretion of exosomes (red dots) that mediate motility and adhesion of tumor cells to the underside of polycarbonate filters, over the 18 h period as shown. After 18 h (**B**), cells remaining on the upper wells are removed using cotton swabs, and the cells attached on the underside are fixed in 4% formalin, stained, and photographed.

As a cell attachment protein, it is widely believed that there are cell surface receptors for extracellular fetuin. A couple of studies have suggested that cell surface annexins were the putative receptors for fetuin-A [30,31]. However, two lines of experimental evidence painted a different picture. Firstly, when the lower chambers of Boyden motility plates were coated with fetuin-A, washed and coating solution was replaced with fresh serum-free medium, and cells were placed on the top chambers in serum free medium, after about 18 h the cells penetrated the polycarbonate filters, and attached and spread on the underside of the filters [32], as indicated in Figure 2. In other words, it was not necessary for the cells to directly contact immobilized fetuin-A for attachment to take place; fetuin-A acted as a chemoattractant. We have therefore hypothesized that fetuin-A is endocytosed by the tumor cells, and while inside the cells, it modifies and enhances the secretion of exosomes, which, after secretion to the extracellular milieu, promote cell spreading and adhesion. In the presence of fetuin-A (either synthesized by the cells or available in the media), the cells have a higher propensity for attachment and spreading [33]. In the absence of fetuin-A, the cells that do not synthesize and secrete fetuin-A spread poorly, and adhesion and attachment is attenuated [30]. This property of fetuin-A requires sialic acid residues. Asialofetuin-A (fetuin-A denuded of sialic acid residues) lacks the capacity to promote adhesion and chemotaxis [33,34]. For this reason it serves as a negative control just like BSA which also lacks the capacity to support adhesion and chemotaxis of tumor cells. To support this hypothesis, we have demonstrated that attachment and spreading of cells in the presence of fetuin-A is attenuated whenever the secretion of exosomes is halted, such as when intracellular calcium is buffered using BAPTA-AM [35], or when the uptake of cellular exosomes is inhibited by heparin [36]. These studies suggest that the fetuin-A-mediated cell attachment is indirect and involves cellular exosomes [37].

## 4. Potential Role of Fetuin-A in a Bone Tumor Microenvironment

Apart from the blood, fetuin-A is highly concentrated in the bone [38]. Interestingly, bone is a favored site for colonization by tumor cells such as breast, prostate, and lung [39]. The bone tumor microenvironment or metastatic niche is a very challenging research frontier in cancer research, because the tumors that colonize it are practically incurable [39]. Tumor cells that colonize the bone expand their niches by activating the resident osteoclasts to break down bone. For this reason, breast cancer [40,41] and multiple myeloma [42] are regarded as osteoclastic tumors because the activities of osteoclasts predominate over those of osteoblasts. The mineral component of bone is hydroxyapatite, which has a strong affinity for fetuin-A [43], and so it represents a microenvironment where fetuin-A is concentrated. Based on the observation that fetuin-A is a chemoattractant [32,44], it is conceivable that it is one of the key molecules that attracts tumor cells to the bone metastatic niche. Interestingly, it has been reported that prostate cancer cells that colonize the bone synthesize and secrete ectopic fetuin-A [45]. Apart from promoting the growth of the tumor cells in the bone, fetuin-A may also slow down the formation of new bone during bone remodeling, given the high concentration of calcium and phosphate in the micro-environment [8]. In addition, fetuin-A may stabilize and maintain the activity of matrix metalloproteinases [46] to break down collagen, the scaffold on which hydroxyapatite is deposited to expand the niche as the tumor cells grow.

## 5. Role of Fetuin-A in Tumor Cell Growth, both in vitro and in vivo

Fetuin-A is predominantly synthesized and secreted into the blood by liver parenchymal cells, maintaining the serum concentration at approximately 0.5 mg/mL [47]. It has generally been assumed that since the concentration of fetuin-A in the blood is relatively high, low levels of fetuin-A synthesized by tumor cells are irrelevant. Moreover, it has been known since the establishment of tissue culture that whereas most transformed and untransformed cells require serum for their growth in vitro, some tumor cells have little or even negligible requirement for serum [48,49]. Assuming that fetuin-A is the major attachment and growth factor in serum as has been alluded to above, we and others questioned whether fetuin-A had a role in tumor cell growth and progression in vivo. We injected Lewis Lung Carcinoma (LLC) cells (tail vein and orthotopically) into syngeneic C56\BL6 mice that were either fetuin-A wild-type, heterozygous, or null (*Fet^+/+^*; *Fet^+/−^*; and *Fet^−/−^*), and monitored tumor growth over several weeks. As expected, lung tumor nodules were prominent mainly in the wild-type mice (*Fet^+/+^*), heterozygous mice had roughly half the number of nodules as seen in the wild-type, and the null mice had no nodules after two weeks [34]. The conclusion was that at least in lung cancer, fetuin-A was a major driver of tumor growth in vivo.

On the contrary, another report also appeared at around the same time, demonstrating that intestinal tumors proliferated more in the fetuin-A null mice relative to the wild-type [50]. In this report, the authors argued that at least in intestinal tumors fetuin-A acts as a tumor suppressor. This report prompted us to repeat the study using PyMT transgenic mouse model for mammary tumors that we crossed to *Fet^+/−^* C57\BL6 mice to generate *PyMT+/Fet^+/+^*; *PyMT+/Fet^−/−^*; and *PyMT+/Fet^+/−^* C57\BL6 mice. Again as expected tumor formed rapidly in *PyMT+/Fet^+/+^* and *PyMT+/Fet^+/−^* C57\BL6 mice. Tumor latency was prolonged in the fetuin-A null mice, pointing to the significance of fetuin-A in mammary carcinogenesis [51]. Our data suggested that in mammary tumors, fetuin-A attenuates TGF-β signaling, thereby releasing the breaks that keep mammary tumorigenesis in check [51]. Fetuin-A also attenuated oncogene driven senescence in this model system because there was extensive senescence in the mammary tumor tissues of fetuin-A null, but not wild-type mice [51].

The next question was to determine whether tumor cells could also synthesize their own fetuin-A, or whether tumor growth relied exclusively on the liver-derived protein. We observed that in head and neck squamous cell carcinoma (HNSCC), there appeared to be an increased expression of a higher molecular weight fetuin-A ~70 kDa. The liver-derived human fetuin-A is approximately 49 kDa, depending on the level of glycosylation [52]. We demonstrated that head and neck squamous carcinoma cells do indeed synthesize fetuin-A, which enabled them to proliferate under serum-free conditions in culture, and knockdown of this ectopically synthesized fetuin-A reduced the propensity of these cells to move or invade through Matrigel [53]. We have also demonstrated the ectopic synthesis of fetuin-A by glioblastoma cell lines and tumor tissues [33]. Other investigators have also reported the ectopic synthesis of fetuin-A by pancreatic cancer cells [54,55] and prostate cancer cells [45]. Interestingly, the tumors cells in which ectopic synthesis of fetuin-A has been determined, namely glioblastoma and pancreatic cancer, also happen to be some of the most invasive tumor types [56], suggesting that the glycoprotein plays a significant and yet to be fully appreciated role in tumor progression. For example, the ectopic fetuin-A may promote angiogenesis, particularly in glioblastomas that have elaborate blood supply lines [26]. As previously intimated, our data suggest that one of the key functions of fetuin-A at least in vitro, is to stimulate not only the secretion of exosomes but also their uptake. The role(s) that exosomes play in tumor progression is currently one of the hottest frontiers in cancer research [57,58]. Factors that mediate their secretion and uptake by tumor cells must be defined before we can begin to target them or their content for tumor therapy.

Examination of online public databases (Kaplan-Meier plotter) demonstrate that patients with high ectopic expression of fetuin-A in some cancers such as lung cancer [59], and gastric cancer [60] tend to have lower survival. The poor survival by high fetuin-A expressing tumor cells was highly significant in poorly differentiated gastric tumor cells (overall survival log rank P = 3.6 × 10^-7^) [60]. It would be interesting how the Kaplan-Meir survival curves of prostate and glioblastoma cancer cells of high fetuin-A expressers vs low expressers would look like, since we know that these tumor cells clearly synthesize fetuin-A. It can be extrapolated from these studies that in any given tumor, there will be a subset that synthesize fetuin-A, and that these are likely to be the most aggressive tumor cells, possibly tumor stem cells [61].

## 6. Fetuin-A Autoantibodies and Cancer

Some studies have reported the appearance of auto-antibodies to fetuin-A in cancer patients. Using 2D immunoblot analysis to screen sera from patients with a diagnosis of breast cancer for auto-antibodies reactive to human fetuin-A, Fernandez-Grijalva et al. [62] reported that serum autoantibodies against AHSG could be useful as serum biomarkers for early-stage breast cancer. For the last five years or so, there has been a push to develop accurate diagnostic methods to detect early onset of cancer and autoantibody levels, and those that are directed to AHSG appear quite attractive. For example breast cancer could be detected early using AHSG autoantibodies in patient sera with a sensitivity approaching 79% [63]. More recently, phage display fingerprinting was used to analyze sequentially-acquired serum samples from a patient with advanced prostate cancer. They identified a peptide ligand, CTFAGSSC that increased over time, and concomitantly, the serum antibody reactivity to this epitope also increased in the patient that was being monitored. The antigen mimicking the epitope was identified as AHSG [45]. Thus, it appears that the development of aggressive tumors such as prostate, pancreatic cancer and glioblastoma could be preceded with the appearance of fetuin-A auto-antibodies. Thus in the advent of the era of precision and personalized medicine, fetuin-A auto-antibodies could be one of the earliest indicators of tumor growth and used as a screening tool.

## 7. Concentration of Fetuin-A in the Serum and Tumor Microenvironment during Tumor Progression

For many years, scientists have measured the concentration of fetuin-A in the serum or tumor microenvironment during cancer progression. Most of the earlier studies demonstrated a decrease in serum concentration of fetuin-A, particularly in hematological malignancies as tumors progressed [64,65], but it was not clear whether this decrease was as a result of reduced synthesis by the liver (fetuin-A being a negative acute phase protein), or whether the tumor cells actively consumed the fetuin-A from the micro-environment and ultimately the blood. It is unlikely that tumor cells would add or subtract fetuin-A from the blood to a significant extent. For example, the concentration of fetuin-A synthesized and secreted by tumor cells into the extracellular milieu is much less than the blood levels of ~0.5 mg/mL [33]. However, it is possible that the appearance of modified forms of fetuin-A such as fucosylated forms could become reliable tumor biomarkers for the diagnosis or staging of cancer in the future (Table 1).

## 8. Fetuin-A and Toll-like Receptor 4 

The pattern-recognition proteins (Toll-like receptors, TLRs) interact with a myriad of protein ligands, including fetuin-A, which was recently reported to be a ligand for TLR4, with implications in the etiology of lipid metabolism and diabetes [78,79]. Because of TLR4’s involvement in cancer cell signaling, it is likely that fetuin-A is one of the ligands that signals through TLR4 to drive tumor progression.

## 9. Fetuin-A and Tumor Progression—Take-home Lessons

Fetuin-A (AHSG) has been studied for the past ~74 years, and yet, its potential role in tumor progression is just beginning to be appreciated. What is now clear, however, is that it is a multifunctional protein that is essential for a number of physiological functions ranging from inhibition of ectopic calcification, for which much work has been done, to tumor progression, for which the progress has been much slower. Synthesis of fetuin-A by a subset of tumor cells or even other supporting cells, such as tumor-associated macrophages (TAMs) in the tumor micro-environment, may confer growth and metastatic propensity to the tumor cells. The presence of fetuin-A in the tumor microenvironment may also promote the efficient synthesis, secretion, and endocytic uptake of exosomes, resulting in the promotion of tumor growth. Fetuin-A may also modulate the synthesis and activities of matrix metalloproteinases that are essential for the metastatic spread of tumors. All these salient properties of fetuin-A suggest that a closer look at this serum glycoprotein is a must if our goal is to fully understand the basic mechanisms that govern tumor progression.

## 10. Fetuin-A and Type 2 Diabetes Mellitus

In a case-cohort study of Sajana et al. [80], a significant association between fetuin-A and type 2 diabetes mellitus (T2DM) risk in both males and females was established. This study concluded that higher circulating fetuin-A levels are associated with incidence of T2DM. The study was supported by another which did a systematic search from Medline, EMBASE, PubMed and Web of Science public electronic libraries using fetuin-A and diabetes as key words. Here over 2,000 cases of diabetes were analyzed, and they noted that one standard deviation increment of fetuin-A level was associated with a 23% greater risk of developing T2DM. As a caveat, the association between fetuin-A and diabetes increments seemed more relevant in women. Nevertheless, they concluded that higher circulating fetuin-A levels were associated with increased risk for T2DM [81]. Earlier studies intimated a strong link between fetuin-A and obesity related complications [82]. Interestingly, weight loss, aerobic exercises, metformin, and pioglitazone have individually been shown to attenuate circulating fetuin-A levels [82]. Lastly, a report by Matthews et al., demonstrated that fetuin-A null mice were protected against obesity and were more insulin-sensitive compared to wild-type mice [83]. In the following paragraphs, we will review work that has been done regarding the mechanistic insights by which fetuin-A modulates the progression of T2DM.

## 11. Action of Fetuin-A at the Insulin Receptor

**Figure 3:**
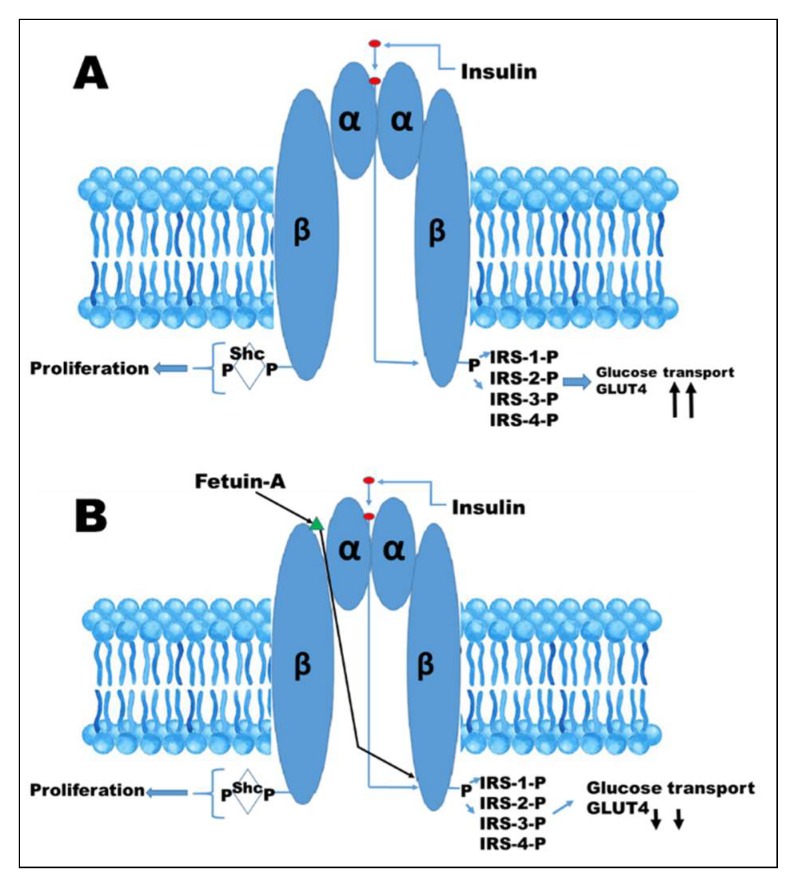
Insulin signaling. (**A**), activation of the insulin receptor by binding of insulin to the α-subunits of its receptor, initiates autophosphorylation of the intracellular portion of the β-subunits, resulting in the recruitment and phosphorylation or receptor substrates such as IRS-1, IRS-2, IRS-3, and IRS-4 Shc proteins. Shc activates Ras-MAP pathway, culminating in proliferation. IRS proteins activate the PI3K-AKT pathway, the signal of which is transmitted to promote translocation of GLUT4 and glucose transport into the cell. (**B**), the binding of fetuin-A to the extracellular portion of beta subunit attenuates tyrosine kinase signaling, resulting in reduced glucose transport and hence a possible source for insulin resistance.

The first report suggesting that fetuin-A is a natural inhibitor of insulin receptor tyrosine kinase was from Dr. Grunberger’s group [84]. In this report, they demonstrated that fetuin-A inhibited insulin-induced tyrosine phosphorylation of the beta subunit of insulin receptor by 40%. In addition, bovine fetuin-A completely blocked insulin-stimulated DNA synthesis in rat hepatoma cells [3]. Studies by Chen et al. [85] on the other hand, using rat adipocytes, demonstrated that fetuin-A inhibited both basal and insulin stimulated phosphorylation of Elk-1 signaling, but they did not observe effects of fetuin-A on insulin-stimulated translocation of GLUT4 or glucose transport [85]. Insulin action begins when it binds to its high affinity cell surface receptor, a hetero-tetrameric protein comprised of two extracellular α-subunits and two transmembrane β-subunits, as illustrated in Figure 3.

Insulin binding to the pocket created by the two α-subunits results in conformational changes that translates into auto-phosphorylation upon the activation of the intrinsic tyrosine kinase of the β-subunit [86]. Activation of the insulin receptor tyrosine kinase leads to the phosphorylation of the insulin receptor substrates (IRS)-1, -2, -3, and -4, as well as Shc proteins [86] (Figure 3). The Shc proteins activate the Ras-MAP kinase pathway, resulting in proliferation of mitogenic events, while IRS proteins mostly activate PI3K-AKT, which mediates insulin’s metabolic effects, including glucose transport [86].

Fetuin-A binds to tandem fibronectin domains within the 194-amino acid residue extracellular section of the β-subunit of the insulin receptor. This binding site is further away from the high affinity insulin binding pocket (Figure 3). Interestingly, the binding of fetuin-A to the β-subunit appears to follow the principle of cooperativity in that binding of insulin to its high affinity site on the α-subunit creates a conformational change that enhances or improves the binding of fetuin-A to the β-subunit [18]. A report by Goustin et al., demonstrated that fetuin-A blocks insulin-stimulated GLUT4 translocation and AKT activation in mouse myoblasts [87]. They also demonstrated the ability of fetuin-A to inhibit the insulin receptor auto-phosphorylation of highly purified insulin holo-receptors in a cell free system [87]. They concluded that fetuin-A antagonizes the metabolic functions (including glucose transport) initiated by insulin receptor activation without interfering with insulin binding (Figure 3).

## 12. Action of Fetuin-A on β-islets Cells of the Pancreas

**Figure 4:**
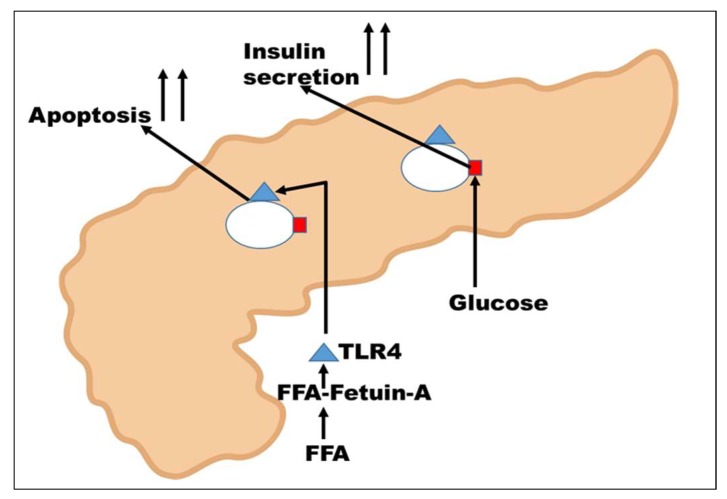
Fetuin-A-mediated insulin resistance in the pancreas. High glucose initiates insulin secretion by the islet cells. Free fatty acids such as palmitate initiate their signals in the infiltrating adipocytes via TLR4, resulting in upregulation of cytokine synthesis, culminating in the apoptosis of islets cells and reduced insulin secretion.

A rise in the blood glucose level is initially sensed by the β-islets cells where glucose is internalized via the cell surface GLUT2 transporters (red square in Figure 4), eventually leading to increases in insulin secretion [88]. Recent studies have indicated a robust cross-talk between fatty pancreas and fatty liver affecting the local inflammation and insulin secretion by the islets cells. In these studies, differentiated adipocytes were detected in the vicinity of pancreatic islet cells [19,79]. Expression levels of cytokines such as IL6 and CXCL8 were elevated by fetuin-A and free fatty acids such as palmitate in a TLR4-dependent manner [19,79,89]. Interestingly, it has been demonstrated that free fatty acids such as palmitate do not bind directly to TLR4 but rather via fetuin-A [78]. In other words, palmitate interacts with fetuin-A, which then links the FFA to TLR4, to induce pro-apoptotic signals in islet cells, as shown in Figure 4. Fetuin-A also impaired glucose-induced insulin secretion in a c-Jun N-terminal kinase (JNK)- and Ca^2+^-dependent manner [19,89]. Interestingly, JNK and other pro-inflammatory cytokines such as TNFα can mediate serine phosphorylation of the IRS proteins associated with the insulin receptor, resulting in its inhibition [90]. Thus, increased secretion of fetuin-A in adipose tissue engages toll-like receptors (TLRs), contributing to pro-inflammatory state leading to insulin resistance and metabolic syndrome [82,91,92]. This is another mechanism by which fetuin-A mediates insulin resistance in T2DM.

## Figures and Tables

**Table 1:** Changes in the levels of fetuin-A (protein or messenger RNA (mRNA)) and post-translational status of the glycoprotein during tumor progression.

Changes in the Levels of Fetuin-A (Protein or mRNA) and Post-Translational Status of the Glycoprotein During Tumor Progression	Reference
Increased fetuin expression in tumor tissue (Protein)	[66,67,68,69]
Increased fetuin expression in tumor tissue (Protein and mRNA)	[33,53,54]
Increased fetuin-A expression in serum	[70]
Increased in malignant pleural effusion of lung cancer	[71]
Increased uptake of fetuin-A by tumor cells	[72]
Increased high mannose glycan structures of fetuin-A in lung adenocarcinoma but not in control normal lung	[73]
Increased fucosylation of fetuin-A in hepatocellular carcinoma and cholangiosarcoma	[74,75]
Increased levels of fetuin-A in CSF of low grade glioma patients	[76]
Reduced levels of fetuin-A in sera of patients with hematological malignancies.	[64,65]
Reduced levels of fetuin-A in the microenvironment during the progression of GI tumors	[50]
Reduced levels of fetuin-A in lung squamous cell carcinoma	[77]
